# Ferrostatin-1 alleviates the damage of C2C12 myoblast and mouse pelvic floor muscle induced by mechanical trauma

**DOI:** 10.1038/s41420-023-01482-2

**Published:** 2023-07-07

**Authors:** Yong He, Guotao Huang, Shasha Hong, Xiaohu Zuo, Zhihan Zhao, Li Hong

**Affiliations:** Department of Gynecology and Obstetrics, Renmin Hospital of Wuhan University, Wuhan, Hubei Province 430060 People’s Republic of China

**Keywords:** Experimental models of disease, Urogenital diseases

## Abstract

Ferroptosis is a special form of regulated cell death, which is reported to play an important role in a variety of traumatic diseases by promoting lipid peroxidation and devastating cell membrane structure. Pelvic floor dysfunction (PFD) is a kind of disease affecting the quality and health of many women’s lives, which is closely related to the injury of the pelvic floor muscle. Clinical findings have discovered that there is anomalous oxidative damage to the pelvic floor muscle in women with PFD caused by mechanical trauma, but the specific mechanism is still unclear. In this study, we explored the role of ferroptosis-associated oxidative mechanisms in mechanical stretching-induced pelvic floor muscle injury, and whether obesity predisposed pelvic floor muscle to ferroptosis from mechanical injury. Our results, in vitro, showed that mechanical stretch could induce oxidative damage to myoblasts and trigger ferroptosis. In addition, glutathione peroxidase 4 (GPX4) down-regulation and 15-lipoxygenase 1(15LOX-1) up-regulation exhibited the same variational characteristics as ferroptosis, which was much more pronounced in palmitic acid (PA)-treated myoblasts. Furthermore, ferroptosis induced by mechanical stretch could be rescued by ferroptosis inhibitor (ferrostatin-1). More importantly, in vivo, we found that the mitochondria of pelvic floor muscle shrank, which were consistent with the mitochondrial morphology of ferroptosis, and GPX4 and 15LOX-1 showed the same change observed in cells. In conclusion, our data suggest ferroptosis is involved in the injury of the pelvic floor muscle caused by mechanical stretching, and provide a novel insight for PFD therapy.

## Introduction

Skeletal muscle is the largest organ of the human body, accounting for about 40–45% of the total body weight. It takes an indispensable role in the body’s mechanical activities and metabolic functions, and is essential for the motion of our life [1]. The pelvic floor muscle (PFM) is a key structure of the pelvic organ support system, dysfunction of which can cause clinicopathological changes, resulting in pelvic floor dysfunction (PFD) diseases, including pelvic floor organ prolapse and urinary incontinence [2]. These diseases have severely undermined the physical health and quality of life of women, brought huge physical and psychological burdens on them, and given rise to enormous economic expense to the medical care system and women themselves [3, 4]. In response to these serious problems, substantial efforts have been devoted to the research of the pathological mechanism of these diseases and seeking more effective treatment measures. Vaginal delivery can lead to pelvic floor dilation and muscle stretching, which would provoke pelvic floor muscle damage and attenuate the support strength of pelvic floor muscle, finally bringing on PFD. Studies have observed that women over-weighted are more likely to develop PFD, and it is unclear whether hyperlipidemia affects pelvic floor muscle status [5, 6]. Therefore, strategies for plerosis of the pelvic floor muscle function are practical for PFD treatment.

Ferroptosis is an iron-dependent regulated cell death, which is first discovered and proposed by Dixon et al. [7] in 2012. This type of cell death is closely related to abnormal iron metabolism and lipid peroxidation. Ferroptosis is morphologically, biochemically, and genetically distinct from other cell death types, such as apoptosis, necrosis, and pyrolysis. In terms of morphology, this pattern of death is mainly manifested as mitochondrial membrane constriction, increased membrane density, and obscured, decreased, or disappeared mitochondrial cristae, but nuclear membrane is intact; in biochemical aspect, it is manifested as increased intracellular iron levels and a large amounts of reactive oxygen species (ROS), decreased glutathione peroxidase 4 (GPX4) activity or expression, and accumulated lipid metabolites [8]. In addition, ferroptosis is regulated by a series of specific genes, including GPX4, lipoxygenase (LOX), Acyl—CoA synthetase long-chain family member 4 (ACSL4), P53 and solute carrier family 7 member 11 (SLC7A11), etc., and can be specifically inhibited by ferrostatin-1 and liproxstatin [9, 10].

Mechanical stress can evoke the accumulation of reactive oxygen species. Normal mechanical strain induces a physiological increase in ROS levels, while excessive mechanical stress will lead to excessive ROS accumulation and ultimately lead to oxidative damage to tissues, such as skeletal muscle and vascular smooth muscle [11–13]. Oxidative damage has been reported to be involved in PFD caused by mechanical trauma [14, 15]. Ferroptosis is a kind of iron-dependent oxidative cell death, which leads to the dysfunction of the antioxidant system, resulting in the loss of redox homeostasis and cell death [16, 17]. Recently, it had been reported that 15-lipoxygenase 1(15LOX-1), a marker of ferroptosis, was increased after mechanical injury of mouse cerebral cortex, and consistent with this, GPX4 decreased was observed. However, using baicalein (12/15LOX inhibitor) could significantly reverse the effect of ferroptosis and improve mice spatial memory ability of mice [18]. At the same time, by creating a mouse cortical contusion model, Xie BS et al., found that iron and ROS in cortical cells accumulated, abnormal iron metabolism occurred, and genes related to ferroptosis were up-regulated [19]. Interestingly, however, the expression level of GPX4 was not decreased, but a decrease in glutathione peroxidase activity is observed. Ferrostatin-1 inhibited ferroptosis and significantly reduced the lesion and improved long-term motor and cognitive function [19]. These two studies further prove that ferroptosis partly participates in tissue mechanical injury, but its role in PFD caused by pelvic floor muscle injury is still unclear.

Here we report that ferroptosis-related peroxidation gets involved in pelvic floor muscle injury suffering from mechanical stretching. After mechanical injury, we increased the cellular 15LOX-1 protein and decreased GPX4 protein, with large amounts of peroxide product malondialdehyde (MDA) accumulation within cells, and leaded cells to decay or even death, while ferrostatin-1 could protect cells from mechanical injury. In C57BL/6 mice after vaginal dilation (VD), we observed significant damage to the pelvic floor muscles and the same 15LOX-1, GPX4, MDA changes as those in cells. Furthermore, we also found that the mitochondrial membrane was condensed and the cristae was blurred, even vanished, but the nuclear membrane was intact. Both in vivo and in vitro results indicate that mechanical stretching leads to ferroptosis of the pelvic floor muscle. Additionally, we showed that high-fat leaded to PFM more susceptible to ferroptosis from mechanical stretch, and mechanical injury caused a more significant change in 15LOX-1 up-regulation, GPX4 down-regulation, and MDA accumulation.

We conclude that mechanical stretch causes activation of ferroptosis and further leads to intracellular membrane lipid peroxidation, resulting in PFM damage and PFD finally. Moreover, high fat increases the susceptibility of PFM to mechanical injury-induced ferroptosis, which may explain why obese women are more likely to develop PFD.

## Results

### Mechanical stress decreases cell viability and increases intracellular ROS level and cell death

**Fig. 1:**
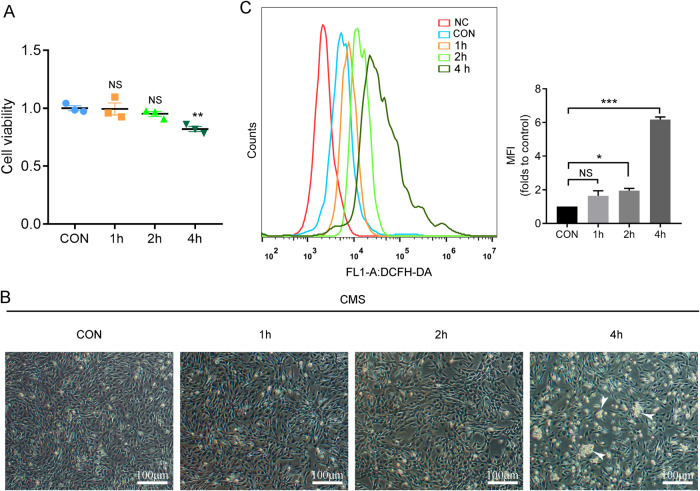
Mechanical stress induces C2C12 injury and cellular oxidative damage. C2C12 myoblasts were treated with CMS at 5,333 μ, 1 Hz for 1, 2 and 4 h, respectively, CON without CMS. **A** Cell viability was measured by CCK-8. **B** Representative image of C2C12 cells treated with CMS, the dead cell clusters were indicated with white arrows. **C** Reactive oxygen species (ROS) was indicated by DCFH-DA with flow cytometry. NC, negative control. CON, control. MFI, mean fluorescence intensity. A, C (right) Data are expressed as the mean ± SEM. NS, no significance, ^*^P < 0.01, ^**^P < 0.05, ^***^P < 0.001 (*n* = 3/group).

Mechanical stretching is essential for the survival, growth, and apoptosis of skeletal muscle cells and has a two-way effect on the vitality of muscle cells. Appropriate mechanical stretching can stimulate a series of reactions in cells and promote cell proliferation or differentiation [20, 21]. On the other hand, excessive mechanical stretching may also cause obvious damage to skeletal muscle and even cell death [22]. Our research found that CMS for1h, 2 h, and 4 h at 1 Hz and 5333 μ (4 mm), respectively, C2C12 cell damage could be initiated at CMS 4 h, resulting in decreased cell viability and increased cell death clusters (Fig. 1A, B). Excessive accumulation of ROS will damage the cell structure. CMS for 2 h and 4 h make ROS accumulate in the cell, but it is more significant at 4 h, which may be a factor leading to decreased cell viability and death (Fig. 1C). Therefore, the follow-up experiment only selects 1 Hz frequency, 5333 μ, 4 h for CMS.

### Mechanical stress results in iron, protein and MDA changes consistent with ferroptosis

**Fig. 2:**
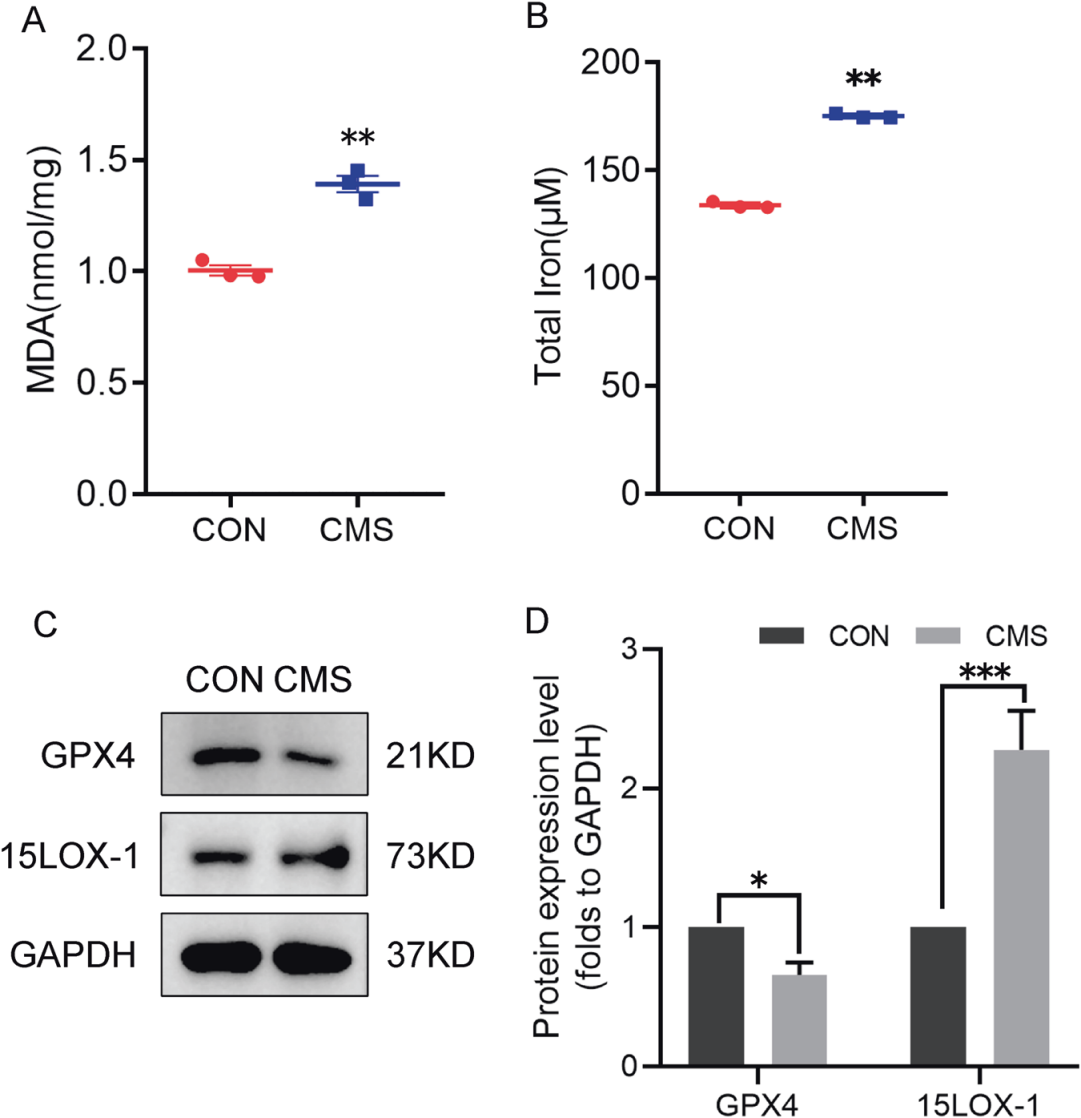
Mechanical trauma induces intracellular lipid peroxidation and triggers ferroptosis. **A** Intracellular MDA, the product of lipid peroxidation, was determined by a MDA Assay Kit. **B** Total iron of cells was detected with an Iron Assay Kit after CMS for 4 h. **C**, **D** The representative two markers of ferroptosis were analyzed by western blotting and its quantitative analysis after C2C12 CMS for 4 h. CON, control. CMS, cyclic mechanical strain. **A**, **B**, **D** Data are expressed as the mean ± SEM. ^*^P < 0.01, ^**^P < 0.05, ^***^P < 0.001 (*n* = 3/group).

Ferroptosis is an emerging type of regulatory cell death [23]. To verify whether the decrease in cell viability and increase in death caused by mechanical stress and the accumulation of ROS are related to ferroptosis, the changes of biochemical characteristics related to ferroptosis in cells after injury were detected. The two important biochemical traits of ferroptosis are the accumulation of iron in cells and the occurrence of excessive lipid peroxidation. Therefore, the total amount of iron in the cells and MDA, the product of lipid peroxidation, were detected after mechanical strain. We found that both of them were increased (Fig. 2A, B). At the same time, GPX4, which specifically scavenges lipid hydroperoxides in the GPX family, was decreased (Fig. 2C, D), and the decreased expression of this protein was considered to be compelling evidence of ferroptosis. Correspondingly, the expression of 15LOX, which promoted lipid oxidation, was increased (Fig. 2C, D).

### Ferrostatin-1 prevented C2C12 myoblast from ferroptosis and the elevation of intracellular iron, as well as ROS, induced by mechanical stress

**Fig. 3:**
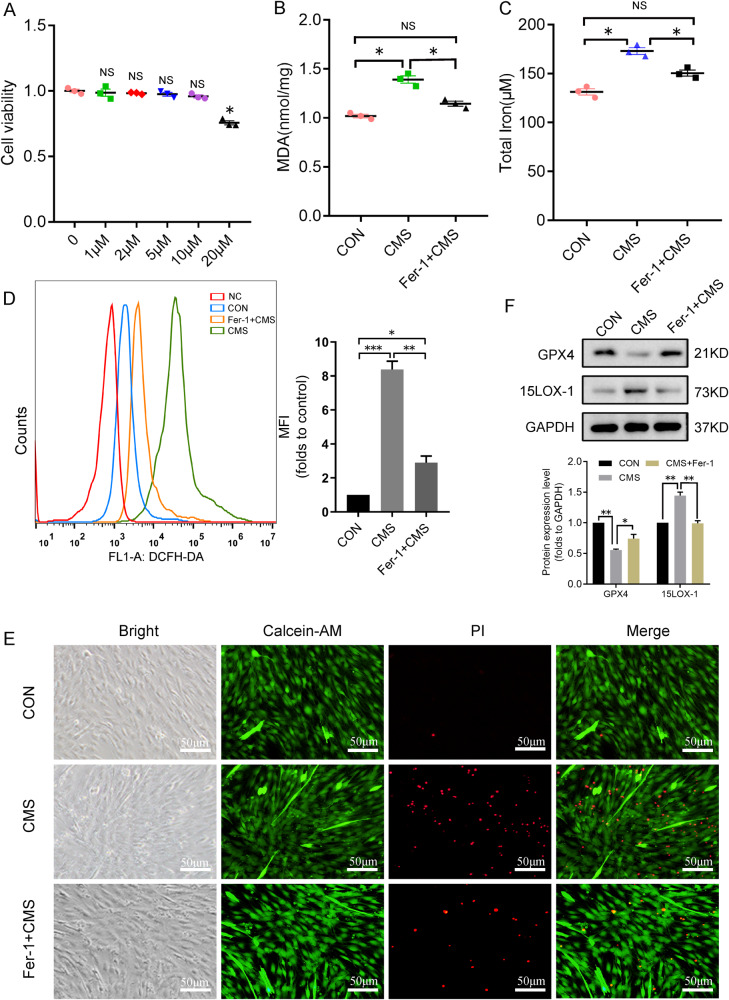
Fer-1 inhibits ferroptosis and injury induced by mechanical stress. **A** C1C12 myoblasts were incubated with various concentrations (up to 20 μM) of fer-1 for 24 h, and the cell viability was measured with CCK-8. **B**–**D** Lipid peroxidation of cells was determined with MDA Assay Kit total cellular iron was detected with Iron Assay Kit and ROS was analyzed by DCFH-DA with flow cytometry after C2C12, which was pretreated with fer-1 for 24 h, CMS for 4 h. **E** The living/dead cells were indicated by Calcein AM/PI staining (Calcein AM, living cells, green; PI, dead cells, red). **F** The representative proteins of ferroptosis were analyzed by western blotting. CON control, NC negative control, CMS cyclic mechanical strain. Fer-1, ferrostatin-1. **A**, **B**, **C**, **D** (right), **F** (down) Data are expressed as the mean ± SEM. NS, no significance, ^*^P < 0.01, ^**^P < 0.05, ^***^P < 0.001 (*n* = 3/group).

Ferrostatin-1(Fer-1) is a specific inhibitor of ferroptosis, which inhibits the production of iron-dependent lipid hydroperoxide, reduces the level of intracellular oxidative stress, and effectively suppresses ferroptosis [24]. In this study, ferrostatin-1 was used to further confirm the role of ferroptosis in cell death induced by mechanical stress. We firstly explored the toxic effects of ferrostatin-1 at 1, 2, 10, and 20 μM on cells, and found that ferrostatin-1 at 20 μM was detrimental on cells, and 0–10 μM had no such effect (Fig. 3A). Therefore, we used 10 μM ferrostatin-1 in the follow-up experiment. After ferrostatin-1 was used, the levels of iron and MDA in the cells after mechanical damage decreased significantly (Fig. 3B, C), and the levels of ROS in the cells also met the same alteration (Fig. 3D). Ferrostatin-1 protected effectively myoblasts from cell death (Fig. 3E). Concurrently, we also found that the decrease of GPX4 expression caused by mechanical damage was also restored by Ferrostatin-1, and the expression of 15LOX-1 was also decreased (Fig. 3F).

### Mechanical stress-triggered ferroptosis in PA-treated C2C12 cells was more intensive

**Fig. 4:**
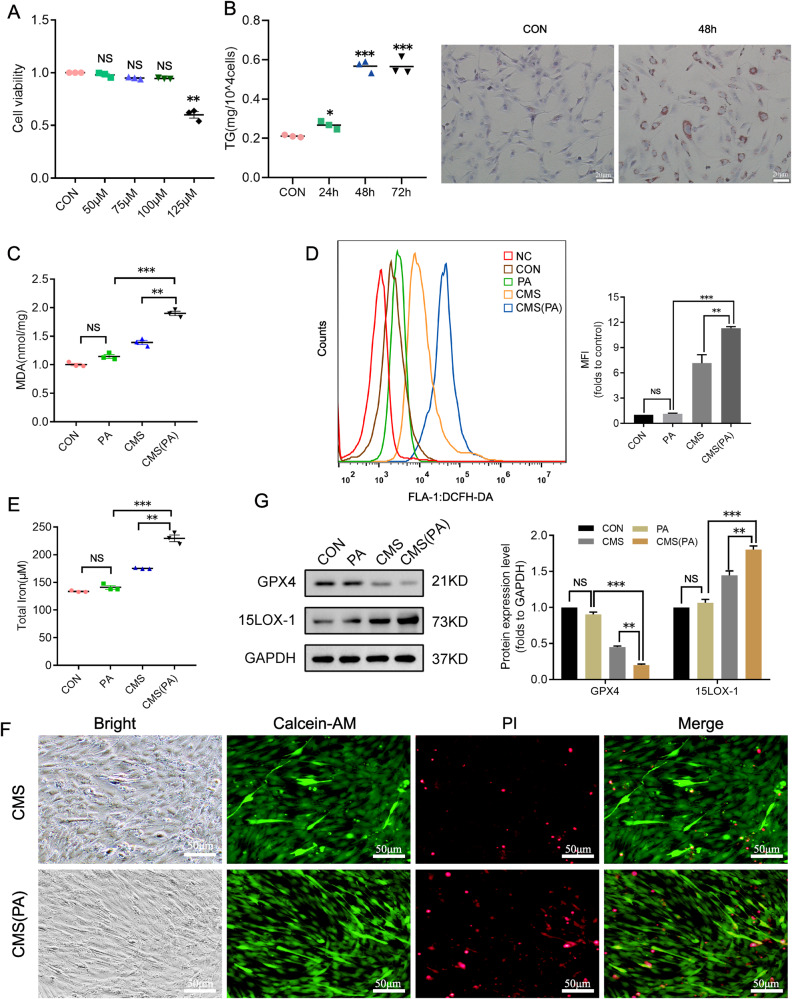
The ferroptosis induced by mechanical stress shows more obvious in PA-treated C2C12. **A** Cell viability was measured after cells treated with 0, 50, 75, 100, 125 μM PA. **B** The effect of PA for cell lipid accumulation was validated by cellular TG determination and Oil Red O staining. **C**–**E** Lipid peroxidation of cells was determined with MDA Assay Kit, ROS was analyzed by DCFH-DA with flow cytometry, and total cellular iron was detected with Iron Assay Kit after C2C12 CMS for 4 h, which was cultured with 100 μM PA for 48 h. **F** the living/dead cells were indicated by Calcein AM/PI staining (Calcein AM, living cells, green; PI, dead cells, red). **G** The representative proteins of ferroptosis were analyzed by western blotting. CON, control, CMS cyclic mechanical strain, PA palmitic acid. **A**, **B** (left), **C**, **D** (right), **F** (down), **G** (right) Data are expressed as the mean ± SEM. NS, no significance, ^*^P < 0.01, ^**^P < 0.05, ^***^P < 0.001 (*n* = 3/group).

Metabolic disorders caused by obesity are the causation of many diseases. Similarly, as an independent risk factor, it plays an important role in the occurrence of female PFD [25]. Therefore, we use 100 μmol/L PA to culture the cells for 48 h to accumulate lipids in the cells and simulate the state of obese cells, without damage to cells (Fig. 4A, B). Subsequently, we applied the same mechanical strain to the PA-treated C2C12 cells, and found that the intracellular ROS level increased significantly after the procedures (Fig. 4D), and the total iron and MDA content also increased (Fig. 4C, E), which were shown to be more significant than that of the cells without PA. Additionally, more dead cells were also observed (Fig. 4F). Whereafter, the expression levels of GPX4 and 15LOX-1 of the treated cells were determined by western blotting, and it was also found that the expression of these two proteins was much higher than that of C2C12 without PA (Fig. 4G). The results indicate that C2C12 cells treated with PA are more susceptible to ferroptosis.

### Similarly, ferrostatin-1 pretreatment had an inhibitory effect on ferroptosis induced by mechanical stress in PA-treated C2C12 cells

**Fig. 5:**
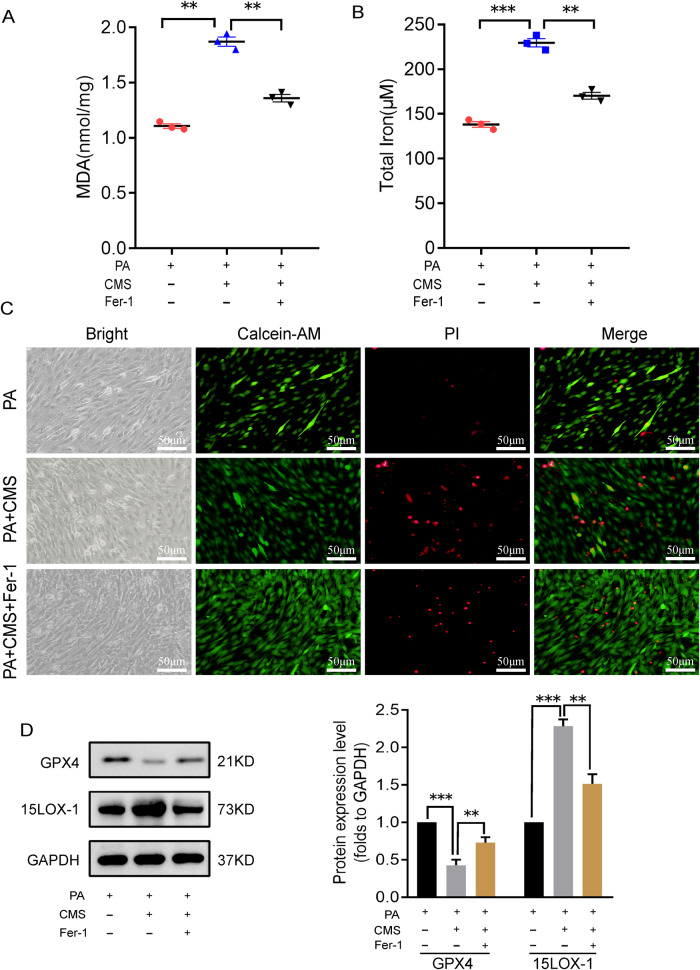
Fer-1 has an identical effect on ferroptosis and injury induced by mechanical stress in PA-treated myoblasts. **A**, **B** C2C12 myoblasts were cultured with 100 μM PA for 48 h and treated with fer-1 for 24 h before CMS for 4 h. After that, MDA were determined by a MDA assay kit (**A**) and total iron by an iron assay kit (**B**) separately. **C** The living/dead cells were indicated by Calcein AM/PI staining (Calcein AM, living cells, green; PI, dead cells, red). D The representative proteins of ferroptosis were analyzed by western blotting. CON, control. CMS, cyclic mechanical strain. PA palmitic acid. **A**, **B**, **D** (right) Data are expressed as the mean ± SEM. NS, no significance, ^*^P < 0.01, ^**^P < 0.05, ^***^P < 0.001 (*n* = 3/group).

Next, we added ferrostatin-1 and cultured the cell for 24 h to observe its inhibitory effect on ferroptosis caused by C2C12 mechanical stretch after PA treatment. Likewise, we found that ferrostatin-1 could effectively mitigate the C2C12 damage caused by mechanical stretch after PA treatment, while reducing the production of intracellular MDA and the accumulation of iron (Fig. 5A, B), as well as reducing cell death (Fig. 5C). Consistent with these, ferrostatin-1 partially rescued the decrease of GPX4 expression, and also prevented the increase of 15LOX-1 expression to a certain extent (Fig. 5D).

### Vaginal dilatation led to pelvic floor muscle damage, mitochondrial shrinkage and ferroptosis in vivo

**Fig. 6:**
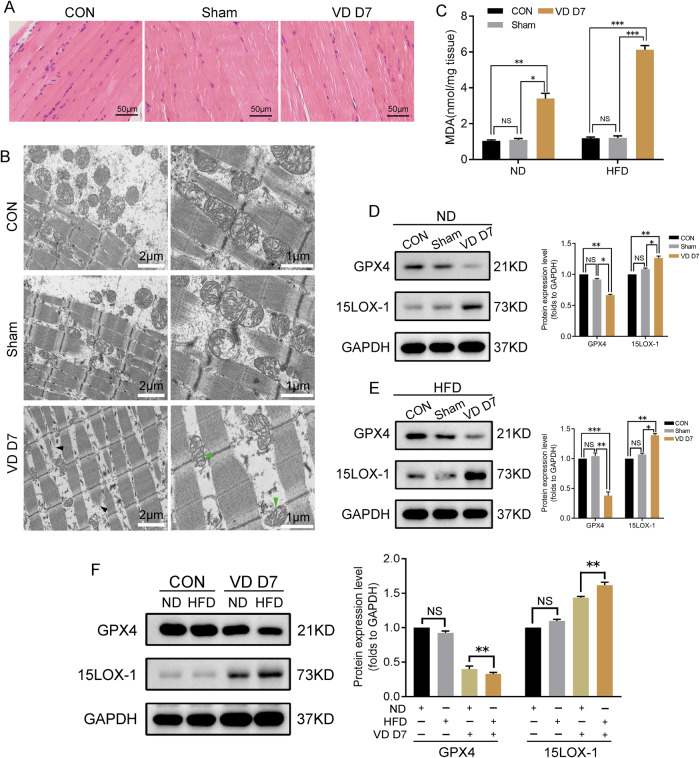
Ferroptosis contributes to PFM injury in vivo. In order to investigate and confirm whether ferroptosis contributes to PFM injury induced by mechanical stretch, a mouse model of pelvic floor muscle stretch was established by VD. On the 7th day after VD, the following tests were performed in each group (*n* = 3/group). **A** HE staining of pelvic floor muscle. **B** Transmission electron microscopy of pelvic floor muscle (damaged muscle filaments, black arrow) and the mitochondrial shrinkage (green arrow). **C** MDA assay was done to determine the lipid peroxidation. **D**–**F** Western blotting was applied to detect the proteins closely related to ferroptosis in the ND group, HFD group, and the two groups were compared. CON, control, CMS cyclic mechanical strain. VD D7, vaginal dilation day 7. ND, normal diet. HFD, high-fat diet. **C**, **D** (right), **E** (right), **F** (right) Data are expressed as the mean ± SEM. NS, no significance, ^*^P < 0.01, ^**^P < 0.05, ^***^P < 0.001.

We established a mouse model of pelvic floor muscle stretch by vaginal balloon dilation. The stretch injury in pelvic floor muscle fibers was confirmed by detection of mitochondrial shrinkage and damaged muscle filament on the seventh day after injury by H&E and TEM (Fig. 6A, B). To verify whether there was ferroptosis in the injured pelvic floor muscle, the MDA in the pelvic floor muscle on the seventh day after the injury was detected. The results showed that the MDA content in the injured pelvic floor muscle was higher than that in the control group and sham group, with no difference between the latter two (Fig. 6C). The changes of GPX4 and 15LOX-1 in the injured pelvic floor muscle were consistent with the features of ferroptosis (Fig. 6D, E), and more distinct in high-fat diet mice than that in normal diet mice (Fig. 6F). In order to observe ferroptosis more intuitively, we performed a TEM on mouse pelvic floor muscle mitochondria. Mitochondrial shrinkage was observed in the pelvic floor muscle of injured mice, which is consistent with the reported ferroptosis mitochondria change (Fig. 6B).

## Discussion

PFD, consisted of a series of benign pelvic floor diseases including pelvic organ prolapse, urinary incontinence, sexual disorders, is common in women. Pelvic organ prolapse (POP) is one of the most common manifestations of PFD in women, which has been confirmed that it comes up with an abnormality in the function of PFM [26]. PFMs are the major part of the nonbony support structure of the posteriorcom-partment, and provide key mechanical support to maintain the correct anatomic position and function of the pelvic organs [2, 27]. Vaginal delivery, obesity, and pelvic surgery are all vital factors that induce PFM damage, leading to POP and other PFDs [5, 6, 28]. Using VD to simulate the vaginal delivery process, we found significant damage to the PFM through H&E staining and TEM, which is consistent with the PFM damage caused by delivery [5].

Ferroptosis is a new form of programmed cell death, which plays an important role in the pathophysiological process of many diseases, such as varieties of tumors [29], neurodegenerative diseases [30] and ischemia-induced organ damage [31], characterized by intracellular iron accumulation, lipid peroxidation and excessive ROS production [2]. ROS is the normal metabolite of almost all tissues including PFM in a physiological state. However, when subjected to pathological stresses such as mechanical damage and energy stress, the overloaded ROS accumulates in cells, which can cause tissue oxidative damage [32]. There have been related reports in PFD [14, 15]. Lipid peroxidation is considered to be one of the most important mechanisms of cell damage under oxidative stress, and has been shown to be related to the pathology of many diseases. Polyunsaturated fatty acids (PUFA) are vulnerable to ROS-induced destruction to produce reactive aldehydes, such as MDA and other similar α, β-unsaturated aldehydes [33, 34]. Just like the pathogenesis of POP, we first found that ROS and MDA increased in mechanically injured myoblasts. Furthermore, MDA in mice PFM after VD met the same change that in cells, suggesting that ferroptosis has an impact on PFD caused by PFM injury. At the same time, we found that the morphology of mitochondria in PFM changed after VD, that is, mitochondria atrophied and cristae blurred, consistent with the typical ferroptosis mitochondrial morphological changes [7].

Glutathione peroxidase (GPXs) is an evolutionarily highly conserved enzyme that uses GSH as a cofactor to reduce peroxide to its corresponding alcohol, thereby limiting the formation of metal-dependent toxic free radicals [35]. GPX4 can act as a phospholipid hydroperoxidase to reduce lipid peroxides to lipid alcohols and remove lipid hydroperoxides [36, 37]. Therefore, GPX4 activity is essential for maintaining lipid homeostasis in cells and preventing the accumulation of toxic lipid ROS, thereby preventing the onset of oxidative iron-dependent non-apoptotic cell death (called ferroptosis) [7, 23]. In our study, it was found that the expression of GPX4 decreased and the expression of 15LOX-1 (an important subtype of LOX, a key regulator of ferroptosis [38]) increased in injured mice and myoblasts. 15LOX-1 is one of the main participants of PUFA enzymatic peroxidation. When iron is oxidized to trivalent iron by peroxide, LOX can bind to molecular oxygen [39, 40]. In the process of ferroptosis, GPX4 may indirectly affect the activity of LOX by reducing cell peroxides, and simultaneously, directly eliminate lipid hydroperoxide to play a protective role.

Iron is another important factor in the occurrence of ferroptosis. The evidence of Dixon et al. [7] showed that iron regulatory protein 2 ((IRP2) controls the iron levels in cells to upregulate ferroptosis caused by erastin. We found that the content of total iron increased and contributed to the accumulation of ROS and MDA, consistent with the previous study [41]. Labile intracellular iron (i.e., free) may be sufficient to catalyze Fenton chemistry on soluble peroxides or lipid peroxides to produce toxic hydroxyl or lipid alkoxy radicals, which can further promote lipid peroxidation, leading to ferroptosis. Meanwhile, iron can also bind to lipoxygenase enzymes, which can generate PUFA hydroperoxides as part of their normal function [42].

Meanwhile, we also observed that ferroptosis triggered by mechanical damage performed more intensely in PA-treated myoblasts and high-fat fed mice, which may be related to the higher lipids of the cells. This may partly explain that obese patients are more prone to suffer from muscle damage [43, 44] and PFD [6]. We also observed that the changes in myoblasts can be effectively inhibited by the ferroptosis inhibitor Fer-1. Fer-1 is the first small molecule inhibitor to be used for ferroptosis, which can prevent erastin-induced cell death [7]. It plays an anti-ferroptosis role mainly by forming complexes with iron, reducing intracellular free iron, and scavenging alkoxy radicals and other toxic products induced by ferrous and lipid hydroperoxides [24].

In conclusion, our study identified that ferroptosis played an important role in the mechanical-induced injury of mice PFM. The occurrence of ferroptosis led to the disequilibrium of redox homeostasis in cells, resulting in excessive accumulation of oxygen free radicals and lipid hydroperoxides, which aggravated the destruction of muscle structure and dysfunction. Pretreatment with ferroptosis inhibitor fer-1 could mitigate the ferroptosis of myoblasts. Combined with the mitochondrial shrinkage of injured pelvic floor muscle in vitro, it indicated that ferroptosis was intently related to muscle injury and PFD. Although more research was needed on the interaction between ferroptosis and pelvic floor muscle injury and PFD, and further basic and clinical evidences are needed to perfect and support our results, our current discovery revealed a novel type of death of injured pelvic floor muscle in the pathogenesis of PFD, and provided a new strategy for the study of PFD.

## Materials and methods

### Mice and study design

Mouse experimentations were carried out by following the institutional guidelines and regulations of Wuhan University. All of the procedures that involved animals were approved by the Institutional Animal Care and Use Committee of Renmin Hospital of Wuhan University and animal care was conducted in accordance with the committee’s guidelines and ARRIVE guidelines. 18 wild-type female C57BL/6 J mice (1-month old) were purchased from the Center for Animal Experiment of Wuhan University. The mice were randomly divided into two groups and raised another 6 weeks in our study: the normal diet (ND) group and the high-fat diet (HFD) group, each group was split into control (CON) group, sham group (treatments are the same as VD group except for vaginal dilation) and vaginal dilation (VD) group. The specific procedures of VD and PFM collection could be seen in our previous study [45]. Animal modeling/treatment and outcome testing were done by different researchers. Mice were housed in the Animal Experimental Center and Institute of Model Animal of Wuhan University during these experiments.

### C2C12 myoblast culture

Immortalized murine C2C12 myoblasts were purchased from Cobioer Biosciences Co., Ltd. (Nanjing, China), and cultured in high-glucose (4.5 g/L d-glucose) Dulbecco’s modified Eagle’s medium (DMEM) (Hyclone, GE Healthcare, Logan, UT, USA) containing 10% fetal bovine serum (FBS) and 1% penicillin-streptomycin in an incubator (HERACELL VIOS 160i, USA) with 5% CO2 at 37 °C.

### Cell mechanical strain

Parameter selection {1 Hz, 5333 μ(4 mm), 4 h} and experimental method are based on our previous study that 5,333 μ strain of cyclic mechanical strain (CMS) for 4 h at a frequency of 1 Hz were the appropriate parameters for the research of mechanical injury [46]. In this study, we first tested these parameters {(1 Hz, 5333 μ, 1 h), (1 Hz, 5333 μ, 2 h), (1 Hz, 5333 μ, 4 h)} and the results showed that C2C12 cell could be damaged at a frequency of 1 Hz with 5,333 μC strain for 4 h. Consequently, we only performed a 5333 μ × 4 h CMS at a frequency of 1 Hz in the next experiment. Firstly, C2C12 cells were cultivated in growth medium. Once the cells reached 70–80% confluence, they were trypsinized and resuspended in growth medium. The cell suspension was diluted 10-fold for cell counting for obtaining a cell suspension with a concentration of 6 × 10^4/ml, and then 2.0 ml cell suspension was cultured on the cell plate. Once the cells reached 80–90% confluence, the cell plate would be placed into the strain loading dish (containing growth medium) to load the CMS. All these cell treatments were independently repeated at least 3 times, and representative numbers and average values were provided.

### Chemicals and reagents

Ferrostatin-1 (Fer-1) was purchased from MedChemExpress (MCE, HY-100579, Monmouth Junction, USA) and dissolved in DMSO (Sigma-Aldrich, D4540) and stored at −20 °C. Other reagent sources are listed below: FBS, trypsin/EDTA solution, DMEM (Hyclone, GE Healthcare, Logan, UT, USA), palmitic acid (PA) (Sigma-Aldrich, P0500), Albumin BovineV (BSA) (Fatty acid free) (Solarbio, A8850); Iron Colorimetric Assay Kit (APPLYGEN, Beijing, China); Lipid Peroxidation MDA Assay Kit, Reactive Oxygen Species Assay Kit, Calcein/PI Live/Dead Viability/Cytotoxicity Assay Kit (Beyotime, Shanghai, China); Oil Red O Stain kit, Triglyceride (TG) Assay Kit (Solarbio, Beijing, China); Cell Counting Kit-8(Dojindo, Japan).

### PA‐induced lipid accumulation in C2C12

The PA was completely dissolved in 75% (v/v) ethanol under heating in a water bath at 55 °C, and then the PA was diluted in DMEM containing 1% (w/v) BSA without fatty acids to prepare a final concentration of 50μmol/ L, 75 μmol/L, 100 μmol/L, 125 μmol/L PA growth medium. The final concentration of ethanol was 0.375% (v/v). The solution was gently shaken for 2 h and then sterilized through a 0.2 μm filter. When the cells on the plate grew to 50–60%, they were replaced with a PA-containing growth medium and cultured for another 48 h. Only 1% BSA and 0.375% ethanol were added to the PA-free control medium.

### Cell viability

The Cell Counting Kit-8 was used for cell viability determination. Generally, the cells were centrifuged and resuspended after being trypsinized from the culture plate, and then transplanting the cell suspension (100 μL/well) in a 96-well plate with at 2 x 10^4/well, before placing the 96-well plate in an incubator for pre-incubation of 0.5–1 h (37 °C, 5% CO2). Next, adding 10 μL of CCK-8 solution to each well. The 96-well culture plate was incubated in an incubator for another 1–4 h, and the absorbance at 450 nm was measured with a microplate reader (PerkinElmer, USA).

### Total iron assay

The Iron Colorimetric Assay Kit was used to quantitatively determine the total iron level in the cells. Wash the cells twice with 2 ml of cold PBS to eliminate the interference of iron in the medium, and aspirate the PBS (if it was not detected immediately, it could be frozen at −20 °C). Cells were lysed with 200 ul/well lysis buffer and placed on a shaker for 2 h before adding iron detection reagent and incubating at 60 °C for 1 h. Finally, Finally, microplate reader was used to measure and calculate the total iron at 550 nm.

### Malondialdehyde (MDA) assay

Cells pooled together were homogenized in 200 μL RIPA lysis buffer (Beyotime, Shanghai, China) while tissue in 500 μL/10 mg using an ultrasonic processor (USA). The homogenates were centrifuged at 12,000 *g* for 10 min at 4 °C and the supernatants were collected. A BCA Protein Assay Kit (Beyotime, Shanghai, China) was used to determine protein concentration. Lipid peroxidation was detected as the reaction of malondialdehyde (MDA) and thiobarbituric acid (TBA) to form a colored complex, and measured at 532 nm with a microplate reader. The level of MDA in cell is expressed as nmol mg^-1^ protein and that in tissue is expressed as nmol mg^-1^ tissue.

### Detection of lipids

Lipid staining was performed with an Oil Red O Stain Kit. In brief, the cell culture medium was discarded to get cells fixed with ORO fixative for 20–30 min after washing twice with PBS. After that, the fixative was discarded and cells were washed twice with distilled water. Afterward, add 60% isopropanol and soak for 5 min and then discard 60% isopropanol and add the fresh prepared ORO Stain for 10–20 min.To make sure there was no excess dye, cells would be washed 2–5 times with water after discarding the dye solution. Next, Mayer hematoxylin was added to counter-stain the nucleus for 1–2 min. Finally, the cells were cover with distilled water and observed under an inverted microscope (Olympus, IX73, Japan). Similarly, quantitative determination of lipids was performed with a Triglyceride (TG) Assay Kit and measured with a microplate reader (PerkinElmer, USA) according to the manufacturer’s instructions.

### Calcein/PI assay for the detection of cell death

The cells were cultured on the cell plate for CMS. After that, the culture solution was aspirated, and the cells were washed with PBS. Then, add 500 μL Calcein AM/PI working solution, and incubate at 37 °C for 30 min. Observation was conducted under an inverted fluorescence microscope (Olympus, IX73, Japan). The nuclei of dead cells were stained with PI (red fluorescence), while the living ones were stained with Calcein AM (green fluorescence).

### Flow cytometric analysis of ROS

Cells were harvested from the culture plate and detected for ROS analysis. Cells were incubated with 2,7-Dichlorodi-hydrofluorescein diacetate (DCFH-DA) for 20 min, washed with FBS-free medium 3 times, and then resuspended in flow buffer. A flow cytometry—fluorescence-activated cell sorting (FACS) Calibur system (BECKMAN COULTER, Kraemer Boulevard Brea, CA, USA) was used to conduct signal collection, and then analyzed with FlowJo software (BD Biosciences).

### Hematoxylin-eosin staining (H&E staining)

The 5-um-thick PFM paraffin sections were dewaxed in xylene, rehydrated through decreasing concentrations of ethanol, and washed in PBS. And then stained with hematoxylin and eosin (H&E). After staining, sections were dehydrated through increasing concentrations of ethanol and xylene.

### Transmission electron microscope (TEM) analysis for mitochondria

On the seventh day after VD modeling, the mouse PFM was isolated and fixed in 4% glutaraldehyde, and then sent to the Ultrapathology Center of Wuhan University People’s Hospital for analysis. Sample observation was performed by TEM (HITACHI HT7700, Tokyo, Japan).

### Western blotting

Western Blotting was conducted following the procedures in our previous research [45]. Briefly, total protein was collecting extracted from mouse PFM or myoblasts with RIPA lysis buffer containing phenylmethanesulfonyl fluoride (PMSF), and then centrifuge for 10 min (4 °C, 12000 *g*, 5 min). Harvesting the supernatant, and determining the protein concentration using a BCA kit (Beyotime, China). Then, the protein loading buffer was added and went through the denaturation at 100 °C for 10 min. Subsequently, 50 μg of total protein from each group was separated on 10% SDS polyacrylamide gels electrophoresis (PAGE), and then transferred to polyvinylidene difluoride (PVDF) membranes. After blocking in 5% skim milk for 1 h at room temperature, membranes were clipped according to the molecular weight of protein and incubated with primary antibodies at 4 °C overnight, and then incubated with the secondary antibody for 1 h at room temperature. Signals were visualized using the ChemiDoc™ Touch Gel Imaging System (BIO-RAD, Cal, USA), and protein expression levels were determined using ImageJ software. The antibody information was as follows: anti-GPX4 (1:500, Proteintech, 67763-1-Ig), anti-15LOX-1 (1:1000, Abcam, ab244205), anti-GAPDH (1:3000, Proteintech, 60004-1-Ig), secondary antibody (1:3000; Proteintech, B900210).

### Statistics

Statistical analysis was performed with GraphPad Prism software version 8 (San Diego, CA, USA). Comparisons between two groups were performed with Student’s *t*-tests (unpaired two-tailed), and one-way analysis of variance (ANOVA) was used for multiple comparisons in three or more groups. *P* value < 0.05 were considered significant. All experiments were performed at least in triplicates.

## Supplementary information

Full length western blots

## Data Availability

The corresponding author will share the data underlying this article on reasonable request.
