# Supplementary figures and images for: Ferrostatin-1 alleviates the damage of C2C12 myoblast and mouse pelvic floor muscle induced by mechanical trauma
Source: Cell Death Discov. 2023 Jul 7;9:232. doi: 10.1038/s41420-023-01482-2 (PMC10328917; doi:10.1038/s41420-023-01482-2)

Figure2 C

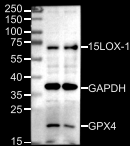

Figure3 F

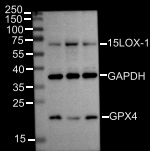

Figure4 G

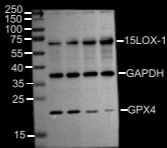

Figure 5 D

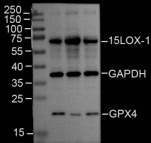

Figure6 D

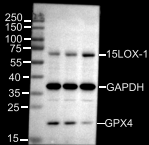

Figure 6 E

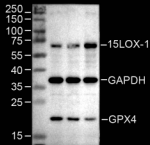

Figure 6 F

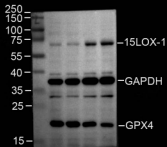

Supplement: Supplementary file 1 — Full length western blots [file 41420_2023_1482_MOESM1_ESM.pdf]
